# Responses of gut microbial community and metabolic function to disposable face mask of *Zophobas atratus* larvae

**DOI:** 10.1007/s44307-025-00092-6

**Published:** 2026-01-04

**Authors:** Chunlan Mao, Kunyue Zhang, Mamtimin Tursunay, Jing Ji, Xiangkai Li

**Affiliations:** 1 State Key Laboratory of Ecological Safety and Sustainable Development in Arid Lands, Northwest Institute of Eco-Environment and Resources, Chinese Academy of Sciences, Lanzhou, 730000 China; 2 Lanzhou Eco-Agriculture Experimental Research Station, Lanzhou, 730000 China; 3 Ministry of Education Key Laboratory of Cell Activities and Stress Adaptations, School of Life Science, Lanzhou University, Lanzhou, China

**Keywords:** Disposable face mask, Biodegradation, Gut microbiome, *Zophobas atratus*

## Abstract

With the prevalence of epidemics, disposable face masks have been used in large quantities and has caused global environmental pollution concern. The gut microbiome of *Zophobas atratus* larvae showed great potential for plastic degradation. In a preliminary study, the larval gut microbiome could degrade masks, which has not been previously reported. This study validated the ability of the gut microbiome to degrade masks. Functional microbiomes and metabolic pathways associated with the degradation of masks were also analyzed. Our findings confirmed that the larvae have high masks-degrading ability with a consumption of 60 ± 0.04 mg/d (dry mass by per 50 larvae), which is gut microbiome-dependent. At the genus level, *Hafnia* and *Corynebaterium* were highly abundant and contributed to masks degradation. The degrading metabolites were then identified, of which 46 were significantly upregulated. Steroid hormone biosynthesis and the cytochrome P450 pathway may be linked to DFM (PP) oxidation and degradation. Finally, *Stenotrophomonas* sp. strain M212 with a masks-degrading ability was screened from these functional microorganisms, further establishing the role of the gut microbiome.

**Supplementary Information:**

The online version contains supplementary material available at 10.1007/s44307-025-00092-6.

## Introduction

Disposable face mask (DFM) is an important method for preventing and controlling respiratory diseases. Especially, during the COVID-19 pandemic, DFM played an irreplaceable role in disease prevention. Meanwhile, demand for DFM increased dramatically. However, large amounts of DFM wastes generated inevitably. An estimated 12 billion DFM were discarded each month (Hekimoglu et al. 2021; Jung et al. 2021; Prata et al. 2021; Oginni 2022). The results of a survey conducted by Global Market Insights forecast the global mask production and market size between 2022 and 2030 based on data collected over the past few years. The market size was reported to 16,631.4 million dollar in 2021 and was expected to reach 11,245.7 million dollar by 2030, growing at compound annual growth rate of 3.3% from 2022 to 2030 (Wang et al. 2023b). The mask industry was expected to grow at a rate of 30% in North America, a region with increasing levels of contamination from masks (Amuah et al. 2022). Therefore, the using of DFM still remains a higher level.

It is worth noting that DFM is mainly made of polypropylene (PP) plastics. The discarded DFM pose serious environmental and ecological hazards. For example, it showed a severe impact on animals, leading to entanglement and eventual death (Du et al. 2022). It was also a huge potential source of environmental microplastics (MPs), which carried radionuclides and antibiotics in freshwater and seawater systems, causing serious water pollution (Lin et al. 2022) and multi-organ toxicity in humans. Additionally, the discarded DFM was a significant source of heavy metals, it released leachable metallic substances, such as copper, cadmium, lead, and antimony (Duan et al. 2020; Ma et al. 2021, 2023; Prata et al. 2021; Ray et al. 2022) and caused metal pollution. Therefore, properly disposing of discarded DFM requires extensive attention. Incineration and landfill are the typical disposal method of medical waste. However, they have been identified as hazardous and contributing to adverse environmental impacts (Emenike et al. 2022; Ray et al. 2022) when treating DFM waste. Though recycling and reusing via mechanical, thermochemical, and chemical processes could convert DFM waste into new products of improved quality or higher environmental value, they needed high energy consumption. Moreover, the recycling of waste faced significant challenges due to the presence of antibiotic resistance genes, bicarbonate bacteria, and pathogens in used masks (Chen et al. 2021; Hui Li et al. 2022; Li et al. 2022). Differently, microbial degradation is recognized as an environmentally friendly and cost-effective approach for treating recalcitrant and emerging pollutants, like microplastics. The biodegradation was facilitated by associated microorganisms and enzymes (Yang et al. 2015a; Peng et al. 2019; Kim et al. 2020; Jeon et al. 2021; Chen et al. 2022) with high treatment efficiency. Consequently, considering the DFM waste mainly composed of PP plastic, microbial degradation is a viable solution.

In recent years, the gut microbiome of some insect larvae, such as Coleoptera (Tenebrionidae, Lepidoptera) and other macroinvertebrates shows great potential in plastic degradation, including polystyrene (PS), polyethylene (PE), PP, polyvinyl chloride (PVC), and polyurethane reactive (PUR) (Yang et al. 2015a, 2021a; Peng et al. 2020; Bae et al. 2021; Brandon et al. 2021; Cassone et al. 2022). Previous studies have shown that consumption of PS by *Tenebrio molitor* (*T. molitor*) and *Zophobas atratus* (*Z. atratus*) was 0.07 and 2.78 mg/larva/day, respectively, and that consumption of plastic was greater than, and microbial-dependent biodegradation of, PS by *Z. atratus* (Wang et al. 2022a). It also reported that the larvae gut microbiome of *T. molitor* could degrade DFM waste (Wang et al. 2023a). Recent studies showed that *Z. atratus* larvae were capable of extensive or limited depolymerization/degradation of PP plastic depended on gut microorganisms (Yang et al. 2021d). However, the biodegradation/depolymerization of DFM has not been characterized. In a preliminary study, we found that *Z. atratus* larvae could consume DFM, suggesting that these masks could be degraded. However, the efficiency of degradation and the gut microbiota remain unknown.

In this study, we hypothesized that DFM could be degraded by the gut microbiome of *Z. atratus* larvae. Therefore, we (1) investigated the degradation of DFM by *Z. atratus* larvae, (2) compared the degradation performance of different layers, (3) characterized the larval gut microbial community and metabolic products using 16S rRNA and non-targeted metabolomics, and (4) screened degrading strains.

## Materials and methods

### Z. atratus and DFM

Biodegradation-tested *Z. atratus* larva (average weight 27.13 ± 0.5 mg/larva) were purchased from Yantan Flower and Bird Market, Lanzhou, Gansu Province, China. Wheat bran (WB) was purchased naturally and without additives from the same place. DFM was purchased from China's e-commerce platform (JD.COM), which ranked No. 1 in terms of sales on the platform in 2022.

### Experimental set-up

To evaluate the consumption and biodegradation of DFM by the larvae, DFM (1.00 ± 0.05 g) were cut into irregular 5–6 cm squares and cleaned with airflow. The incubator employed food-grade PP storage containers measuring 18 × 12 × 7 cm^3^. Each incubator housed 50 *Z. atratus* larvae separately, with a reared density of approximately 0.5 larvae/cm^2^. Throughout the rearing period, all incubators were stored in a 150 L constant-temperature and constant-humidity chamber (LHS-150HC-I, Shanghai Yiheng, Shanghai) with temperature control at 25.0 ± 0.5 °C and humidity control at 65 ± 5% (Yang et al. 2020).

Five treatments were prepared based on feeding conditions: starved *Z. atratus*; WB-fed *Z. atratus*; DFM outer-fed *Z. atratus*; DFM middle-fed *Z. atratus*; and DFM inner-fed *Z. atratus*. The initial feed mass was 5.0 g for DFM-fed and WB-fed larvae. Subsequently, the WB-fed group received an additional 5.0 g of WB every 3 days. Residual DFM flakes were weighed every 3 days and DFM consumption was determined based on mass loss during the feeding period, and larval carcasses were removed in a timely manner to minimize larval inter-feeding as well as disease transmission. Larval survival rate (SR) was calculated by counting the survival larvae every three days, namely the % of the survival larvae every three days in the total initial larvae. Consumption rate (CR) was calculated based on the total mass loss of masks consumed by 50 larvae (mg) and the mass loss of masks consumed per larval weight per day (mg/larvae/d). At the end of the experiment, DFM debris remaining on the larval body surface was removed with compressed air, and the larvae were transferred to a clean box for 12–18 h of fecal collection. The larval feces samples were stored at 4 °C for further analysis. All tests were conducted using the triple method (Yang et al. 2021d). Additionally, five larvae from each group were randomly selected to extract gut tissues and gut contents, which were stored in 2 mL lyophilized tubes, sealed, and labeled in a −80 °C refrigerator.

### Antibiotic suppression test

A Gentamicin inhibition test was conducted to examine the role of larvae gut microorganisms in degradation, following established procedures. WB was treated with gentamicin sulfate (30 mg/g bran) and used to feed the larvae (100 *Z. atratus* larvae per group) for 10 days. These fed larvae were given DFM for 12 days. Ten larvae were randomly selected from each incubator on days 0, 5, and 10. The number of gut bacteria was determined using the colony counting method on Tryptic Soy Agar (TSA) medium. Larval guts were dissected, decontaminated with 75% ethanol, and rinsed with sterilized water. Gut contents were extracted and suspended in 5 mL of sterile saline, diluted in a series from 10⁻^1^ to 10⁻⁷, and incubated on non-selective TSA plates at 37 °C for 24 h (Yang et al. 2015b). To maintain continuous inhibition of gut microbes, DFM was fed alternately with gentamicin-treated bran. And, the frass was collected for analysis.

### DFM degradation analysis

DFM degradation for outer, middle, and inner layers was determined by analyzing water contact angle (WCA), surface morphology using a scanning electron microscope (SEM–EDX), and thermal analysis using a Thermogravimetric analysis (TGA). Mask and fecal samples from mask-fed larvae were analyzed using Fourier-transform infrared spectroscopy (FTIR, NEXUS 670, USA) in the spectral range of 400–4000 cm⁻^1^. Surface chemical components were investigated using X-ray photoelectron spectroscopy (XPS, Axis Supra, Japanese) with scanning over a broadband energy range (0–1200 eV). TGA of DFM and frass samples was performed using a comprehensive thermal analyzer (TG-DSC-DTG, PT 1600, Lindsay, Germany) with a temperature range from 30 °C to 900 °C (Yang et al. 2021b)*.*

### DNA extraction and 16S sequencing

After 12 days of the feeding experiment, 5 larvae from each group were randomly selected for extraction of gut contents. The contents were extracted by immersing the larvae fed with a mask for 12 days in 70% ethanol to anesthetize them and then washed them in sterile saline. The guts were extracted by removing their head and tail with sterilized forceps. The obtained guts were preserved in 2 mL freezing tubes, sealed and labeled, and placed in a −80 °C refrigerator. The larval guts of each group were collected into 1 mL of sterile saline in a 2 mL sterile centrifuge tube. Total DNA samples of the gut bacterial genome were extracted using the FastDNA SPIN kit. The V3-V4 region of the 16S rRNA gene was sequenced by amplicon sequencing. The amplicons obtained after purification were sequenced using 2300 bp paired ends for high-throughput sequencing on the Illumina MiSeq platform at Shanghai Personalbio Biotechnology Co. Filtered reads were spliced into sequences using Fast Length Adjustment of Short Reads (FLASH) with the following criteria: matching length not less than 15 bp, overlap tolerance of 0.1, and removal of reads without overlap. The Sliva database (confidence threshold 0.9) and Blast software were used to annotate sequences with species. Unannotated sequences and test results not part of the analyzed items were removed. The microbial α-diversity was analyzed using Shannon index. The β-diversity was analyzed using Bray–Curtis distances, which were calculated from Hellinger-transformed ASV tables and visualized by Principal Coordinates Analysis (PCoA). For the shift of microbial community, the microbial composition at the phylum and genus levels was analyzed.

### Determination of gut microbial metabolome

For each group, 50 mg of gut content samples were taken in 2 mL centrifuge tubes, and the samples were quickly frozen with liquid nitrogen and then sent to Beijing Biomarker Technologies Co. for metabolite assay analysis. Metabolite extraction was performed by adding grinding beads and 1000 μL of extraction solution containing the internal standard and vortexing for 30 s. The sample was allowed to stand at −20 ℃ for 60 min, centrifuged for 15 min (4 ℃, 12,000 rpm), and 120 μL of supernatant was pipetted into the injection bottle for analysis. The Liquid Chromatography-Mass Spectrometry (LC–MS) data were imported into the metabolomics processing software Progenesis QI for analysis.

### Statistical analysis

Statistical analyses were performed using IBM SPSS statistical software (version 26). ANOVA followed by Student's t-test was used to assess differences in DFM CR, SR, and microbial diversity. Pairwise comparisons between diets and species were conducted using Tukey's correction. Adjusted *p*-values and mean ± standard deviations were reported for all analyses.

## Results and discussion

### Z. atratus SR and DFM consumption

**Fig. 1:**
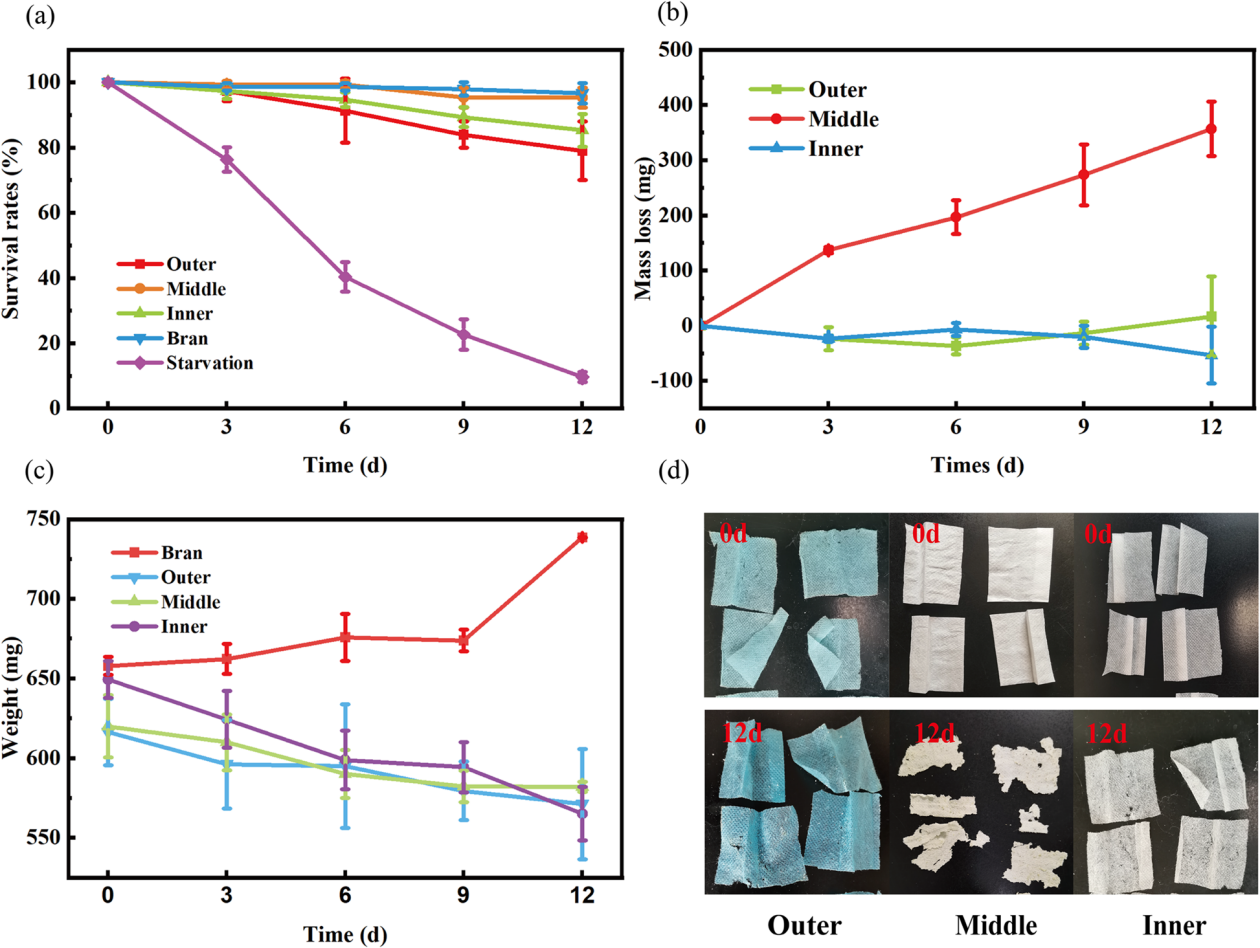
Characteristics of *Z. atratus* larvae feeding consumption. **a** Survival of *Z. atratus* larvae fed different diets for 12 days, (**b**) mass loss (mg) of DFM of *Z. atratus* over 12 days, (**c**) change in body weight of the larvae over 12 days, and (**d**) changes of three layers before and after the larvae feeding

The initial number of larvae fed to each group was 50. At the end of the 12-day experiment, the SR of the bran-fed and starved groups was 96.7 ± 3% and 9.7 ± 1%, respectively (Fig. 1a). The SRs of the outer, middle and inner feeding groups were 79.0 ± 9%, 95.3 ± 3% and 85.3 ± 5%, respectively. The cumulative total amount of middle and outer layers consumed by 50 larvae over a 12-day period was 356.7 ± 49.3 mg and 16.7 ± 7.2 mg, respectively, while the mass of the inner layer increased by 53.3 ± 5.1 mg (Fig. 1b). This was due to the strong adsorption capacity of the DFM, and a large amount of insect frass adsorbed on the surface of the mask resulting in an increase in its mass. After 12 days, larvae in the bran group gained 80.5 mg of weight, whereas larvae in the outer-fed, middle-fed and inner-fed groups lost 45.3 mg, 84.1 mg and 38.0 mg of weight, (Fig. 1c). These results can be explained by the different phenotypic characteristics of larvae utilizing the three layers of DFM (Fig. 1d).

**Fig. 2:**
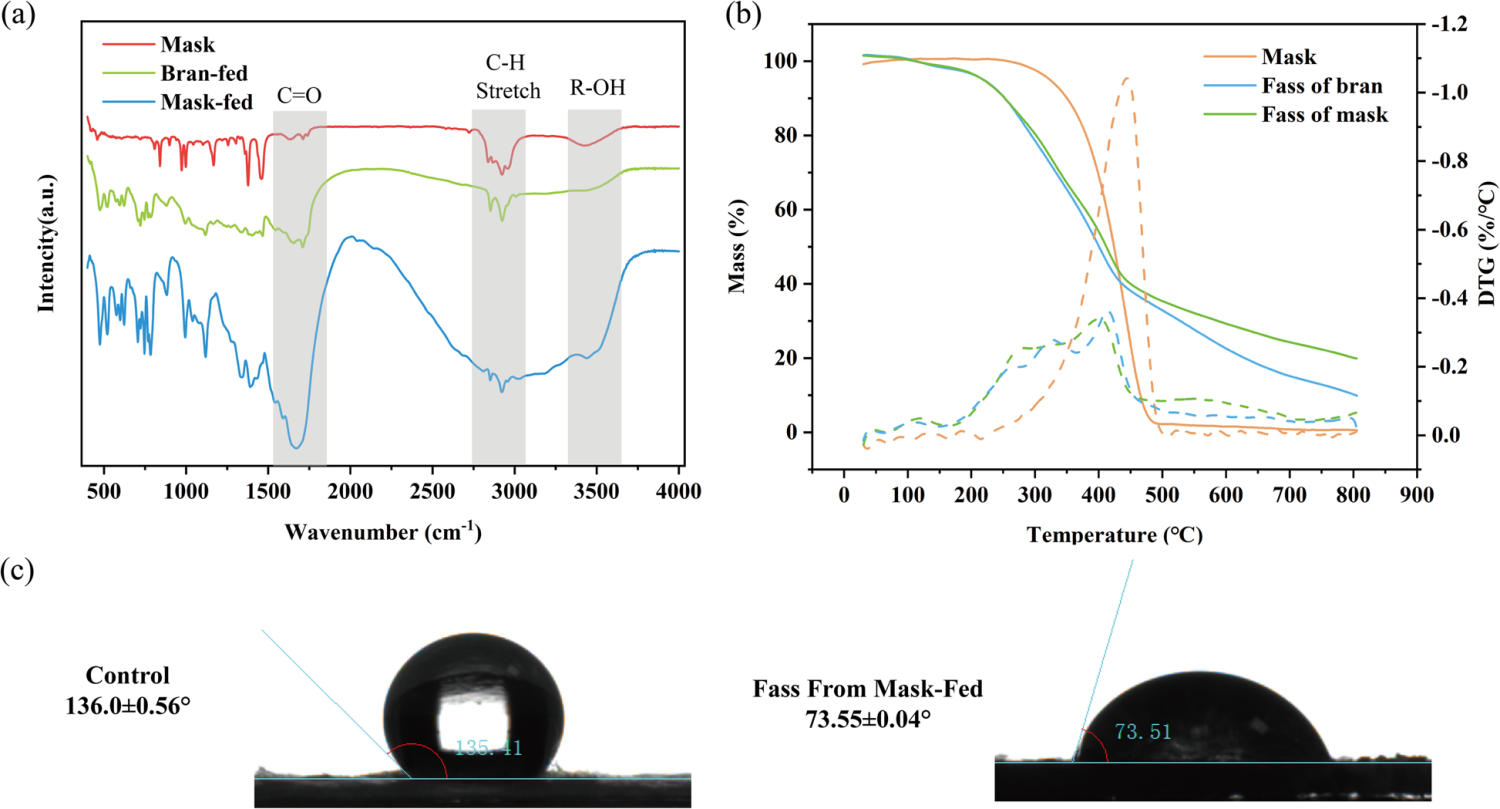
Confirmation of the DFM degradation by *Z. atratus*. **a** FTIR of DMF control, DFM-fed and bran-fed frass of *Z. atratus*; **b** TGA of DFM control, DMF-fed vs. bran-fed frass of *Z. atratus*; **c** WCA of DFM control and DFM-fed frass

**Fig. 3:**
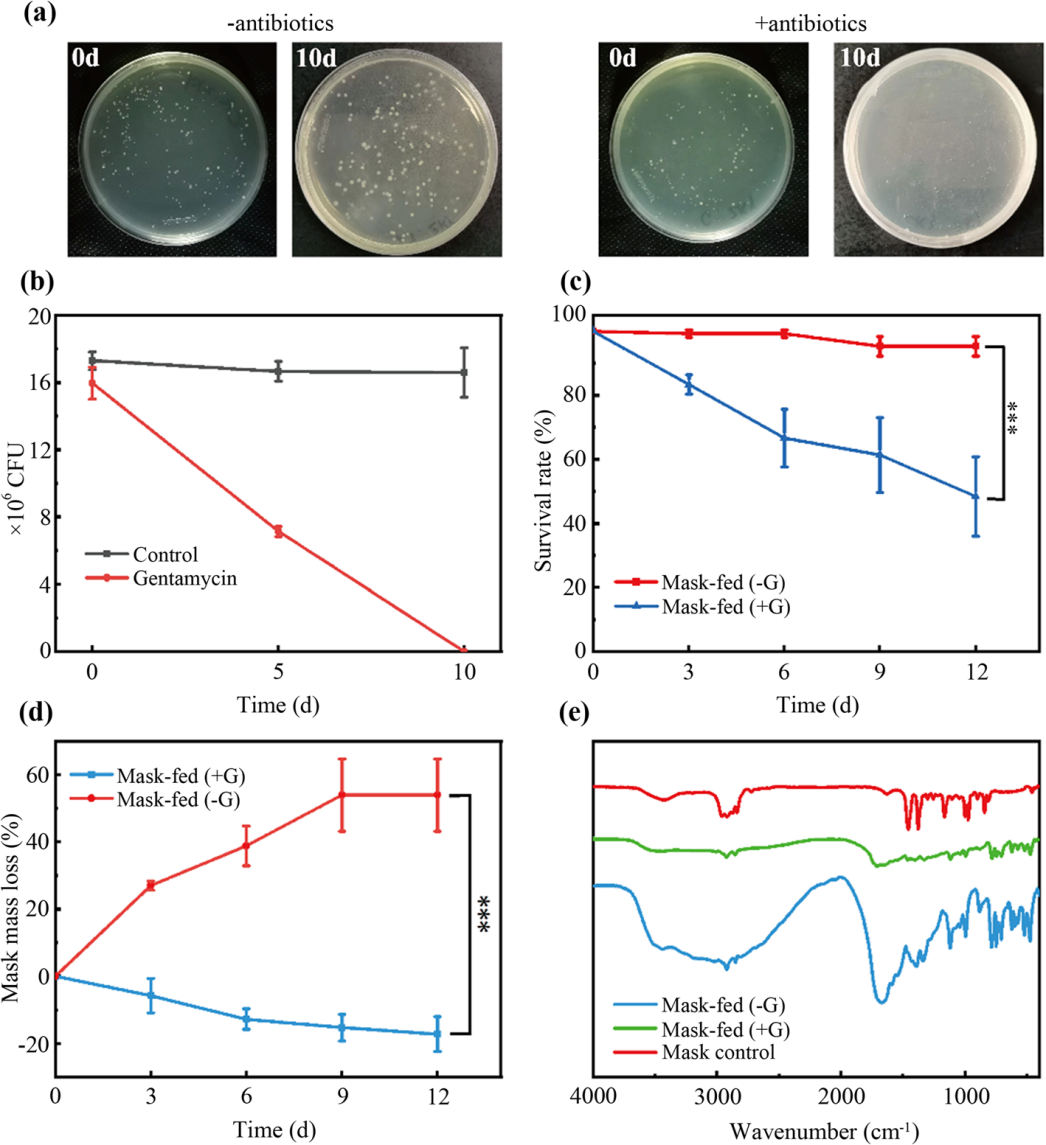
Effects of gentamicin suppression on gut microbiome and DFM consumption. **a**-**b** Changes in colonies without and with gentamicin after 10 days; **c** Survival of *Z. atratus* larvae and (**d**) mass loss (mg/larvae) accumulated by middle layer for 12 days; **e** FTIR spectra of frass samples from DFM-fed and DFM-fed after gentamicin suppression, and the DFM control

Our results showed that *Z. atratus* larvae were selective in feeding on masks and could chew and ingest large quantities of the middle layer of DFM. That was consistent with the results of yellow mealworms feeding on DFM (Wang et al. 2023a). The main reason might be the smaller fiber diameter of the middle layer (Fig. S1), which facilitated feeding and digestion by insects. Mealworms and *Z. atratus* have the same dietary preferences, which are less dense, fluffy-textured plastics. However, reports on feeding about mask or other biodegradable masks were scarce. Compared to the previous studies reporting PP consumption by *Z. atratus*, PP-only-fed larvae also maintained a relatively high plastic consumption of 3.1 ± 0.4 mg/100 larvae/d (Yang et al. 2020, 2021b), similar to our results in the middle layer. The higher SRs of larvae fed on the middle layer were similar to those of larvae fed on WB, suggesting that the DFM middle layer were sufficiently digested and utilized by the larvae. This further indicated that mask degradation occurred. Therefore, the middle layer of DFM was used to investigated the contents showed in Figs. 2, 3, and 7. The decrease in body weight indicated that the single-mask plastic did not provide an adequate source of nutrients, owing to the only hydrogen and carbon in DFM and the lack of nitrogen sources and other nutrients. Although the other two mask layers were fed less, they maintained high survival rates, further suggesting that *Z. atratus* larvae can feed and digest DFM.

### DFM degradation

The bio-oxidation and depolymerization of DFM were investigated using WCA, TGA, and FTIR, which were widely used to study plastic degradation. The FTIR results showed that the chemical bonds of the three DFM layers were similar to that of PP, indicating that the main constituent of DFM was PP. The outer layer had a WCA of 132.22 and a fiber diameter of 21.85 μm, the middle layer had a WCA of 136.54 and a fiber diameter of 1.75 μm, and the inner layer had a WCA of 136.25 and a fiber diameter of 25.82 μm. The WCA results showed that the three DFM layers were highly hydrophobic, and the chemical properties its’ were identical (Fig. S1) (Lin et al. 2022).

Changes in the chemical functional groups between the original DFM (control) and frass of the DFM-fed larvae were determined using FTIR analysis. FTIR spectra results of DFM-fed group exhibited a distinct peak at approximately 1700 cm^−1^ compared to the control, with the peak representing C═O stretching (Fig. 2a). This indicated the oxidation of DFM in the larval gut, suggesting that the debris from the DFM-fed larvae were more hydrophobic than that from the DFM control. By analyzing raw frass, the apparent increase in oxygenated structures provided solid evidence of mask oxidation and depolymerization in the larval gut. The peak at 2500–3500 cm^−1^ represented hydrogen bonding between the hydrophobic and carboxylic acid groups (Peng et al. 2020, 2022b; Yang et al. 2021a). The FTIR spectra of larval frass fed DFM were broader than those of control, indicating an oxygen admixture. This was shown by the appearance of peaks (3000–3500 cm^−1^) associated with R-OH stretching, which was usually associated with monosubstituted benzene derivatives (Bombelli et al. 2017; Yang et al. 2019; Billen et al. 2020; Jiang et al. 2021; Cassone et al. 2022; Peng et al. 2022b).

The TGA indicated that the mask control underwent significant mass loss between 250–500 °C, dropping approximately 85% of the total weight (Fig. 2b), with the maximum decomposition rate occurring at 450 °C. In contrast, no significant mass loss was observed in the TGA curves of frass from mask-fed larvae, which decreased gradually from 30–700 °C with a mass loss of 74.1%. Significant mass loss occurred between 200 and 450 °C, where the decomposition rate was relatively pronounced at 402 °C and peaked. The results showed that the frass contained not only residual DFM samples, but also new organic matter. WB-fed larvae showed a similar trend to mask-fed larvae, with a gradual decrease in mass loss from 30–700 °C, in which two distinct phases and peak decomposition rates observed at 327 °C and 410 °C, respectively. This was similar to what was observed previously in biodegradation of plastics (Peng et al. 2020; Yang et al. 2021a).

Analysis of changes in hydrophobicity of frass surfaces used WCA. The decrease in hydrophobicity contributed to the enzymatic attack on C–C single bonds and vice versa. The WCA of the control mask was 136.0 ± 0.56° (*n* = 3). The value of DFM-fed frass was 73.55 ± 0.004° (*n *= 3), which was significantly lower than that of control samples (*p* < 0.01) (Fig. 2d), suggesting a decrease in the surface hydrophobicity of the mask degradation product. Hydrophobic polymer surfaces inhibited the effective adsorption and catalytic properties of polymer-degrading enzymes. Therefore, the decrease in hydrophobicity contributed to the enzymatic attack on the C–C bond of the mask, demonstrating mask degradation (Brandon et al. 2021).

### Gut microbial community

#### Determination of DFM-degrading ability of the gut microbiome

To clarify the role of the *Z. atratus* gut microbiome in DFM degradation, an antibiotic inhibition assay was performed. Gentamicin sulfate was selected to suppress the gut microbiota because of its activity that against a wide range of bacterial infections, mainly Gram-negative and some Gram-positive bacteria (Yang et al. 2015b). The results showed that the number of gut microorganisms decreased from 16 × 10^6^ to 7.1 × 10^6^ after 5 days. After 10 days, gentamicin inhibition was reduced by two orders of magnitude compared to that in the control group (Fig. 3a, b), indicating that the gut microbiome was effectively inhibited (Yang et al. 2021d). Subsequently, the effect of gentamicin on DFM feeding by larvae was analyzed. Results showed that the SR was 95.3 ± 3.0% in the control group and 48.3 ± 3.4% in the gentamicin-added group. The SR and DFM consumption of the larvae were significantly reduced after the inhibition of gut microorganisms by gentamicin (Fig. 3c, d). The fecal samples were subjected to FTIR analysis. No significant peaks for the inhibition group were observed at approximately 1700 cm^−1^ or 2500–3500 cm^−1^ (Fig. 3e), indicating DFM degradation did not occur. These results suggested that *Z. atratus* larvae lost their ability to oxidize and depolymerize DFM after antibiotic treatment. Further demonstrated the gut microbiome-dependent degradation of DFM by *Z. atratus* larvae. Previous studies have found the gut microbe-dependent depolymerization of PS, PP, and PE in *Z. atratus* (Peng et al. 2020), which was consistent with the results of this study. In addition, *T. molitor* larvae, which belong to the same genus as *Z. atratus*, possessed similar plastic degradation ability. *T. molitor* larvae were capable of digesting lignin, which correlated with the presence of lignin-degrading genes (Cassone et al. 2022; Mamtimin et al. 2023).

#### Microbial community

Sequencing of 16S rRNA amplified fragments in the bran-fed, outer layer-fed, middle layer-fed, and inner layer-fed groups yielded 81229, 85629, 95815, 82203 reads per sample, and 73502, 77737, 86470, 74168 clean reads per sample used for data analysis, respectively. Sequencing results showed 99% coverage of all sequenced samples, indicating that the sequencing results represented the true state of the samples. Observed species in bran-fed, outer layer-fed, middle layer-fed, and inner layer-fed groups were 220, 276, 255, 250, respectively. The aromatic dilution curves of all samples reached a plateau, indicating that the sequencing results were sufficiently reflective of the diversity contained in the current samples, and that increasing the depth of sequencing would not detect the large number of new ASVs/OTUs that had not yet been identified (Fig. S2).

**Fig. 4:**
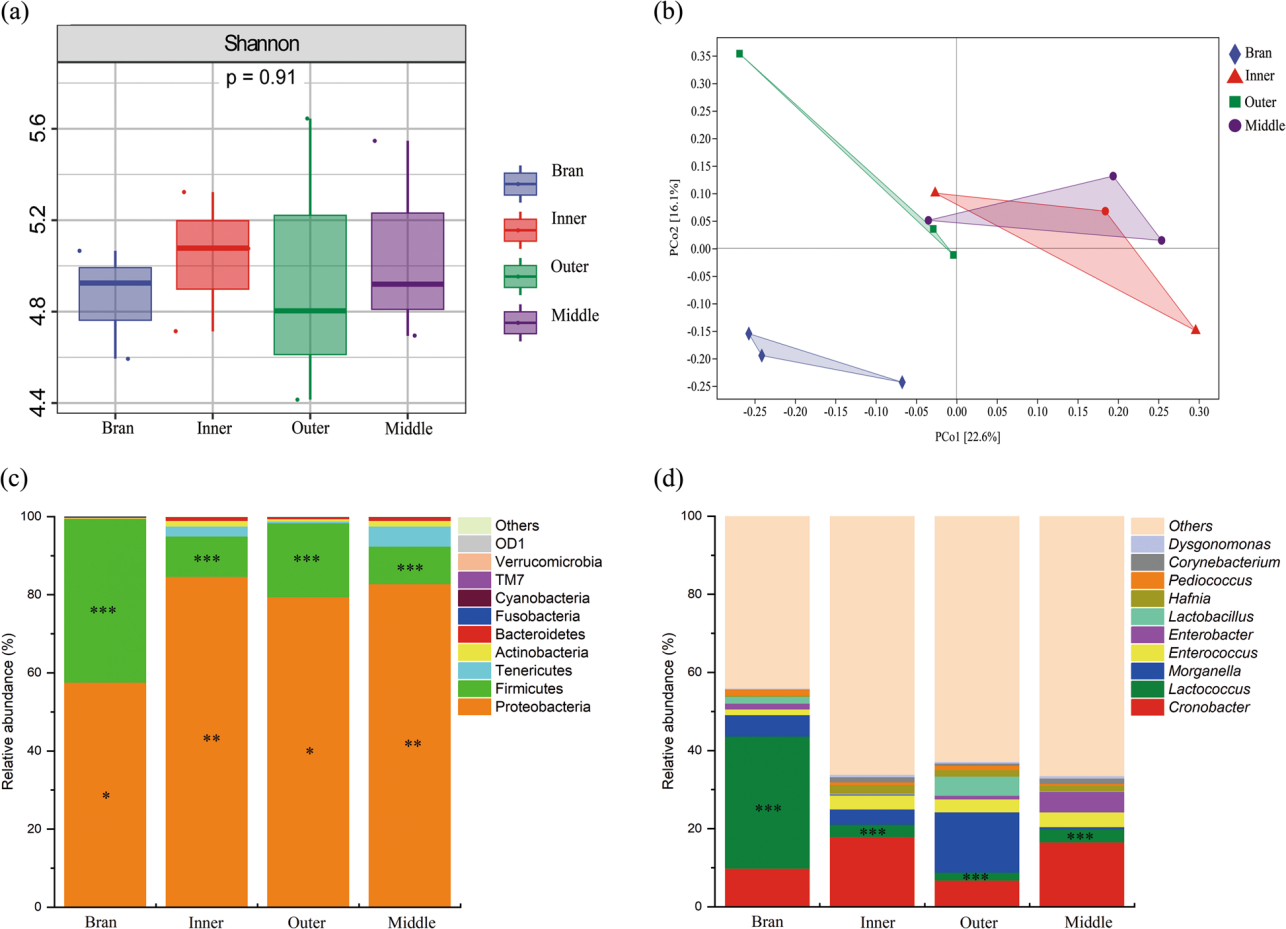
Gut microbial community feeding with different DFM layers. **a** α-diversity using Shannon index, (**b**) β-diversity using PCoA analysis; Changes in gut microbial structure at (**c**) phylum level and (**d**) genus level

The α-diversity showed that the maximum Shannon index was obtained in the inner layer. However, no significant difference between DFM-fed and WB-fed larvae were observed (*p* > 0.05) (Fig. 4a). Considering the β-diversity (PCoA), DFM-fed and bran-fed larvae were significantly clustered, indicating the difference of microbial composition and structure between the groups. However, the three layers of the samples were similar (Fig. 4b). This suggested that DFM affects the gut microbiome and that the resolution/oxidation of DFM was associated with gut microbes.

For the shift of microbial community, each sample showed different sequence distributions at the phylum and genus levels. The major phyla in all the samples were *Proteobacteria* and *Firmicutes* (Fig. 4c). The relative abundance of *Firmicutes* in the bran-fed group was 41.91%, and was 10.34%, 18.95%, and 9.63% in the outer-fed, middle-fed, and inner-fed groups, respectively. *Proteobacteria* was dominant in the outer-fed, middle-fed, and inner-fed groups, increased from 57.57% (bran-fed group) to 84.66%, 79.48%, and 82.76%, respectively. In addition, the relative abundances of *Tenericutes* and *Actinobacteria* also increased in the three-layered samples compared to those in the bran group. At genus level (Fig. 4d), the dominant taxa were significantly different. The top 10 most abundant genera (with a relative abundance threshold of 1%) were further analyzed (Table S1). The dominant bacteria in the bran-fed group were *Lactococcus* (33.80%), *Cronobacter* (9.84%), and *Morganella* (5.49%); *Lactobacillus* (4.91%), *Cronobacter* (6.72%), *Enterococcus* (3.35%), and *Morganella* (15.42%) in the outer group; *Lactococcus* (33.80%), *Enterococcus* (3.79%), and *Enterobacter* (5.26%) in the middle group; and *Enterococcus* (3.55%), *Cronobacter* (17.91%), and *Morganella* (3.90%) in the inner group. *Morganella* and *Lactobacillus* were strongly associated with PP degradation by *Z. atratus* and *T. molitor* (Yang et al. 2021d; Wang et al. 2023a). It was worth mentioning that in the three-layered samples, *Enterococcus* spp. was notably increased compared to those in the bran-fed group. It has been reported to contribute to low density polyethylene (LDPE) degradation (Bombelli et al. 2017; Yang et al. 2019; Peng et al. 2020). The increase of its relative abundance suggested that these genera were significantly associated with mask degradation in *Z. atratus* larvae gut.

**Fig. 5:**
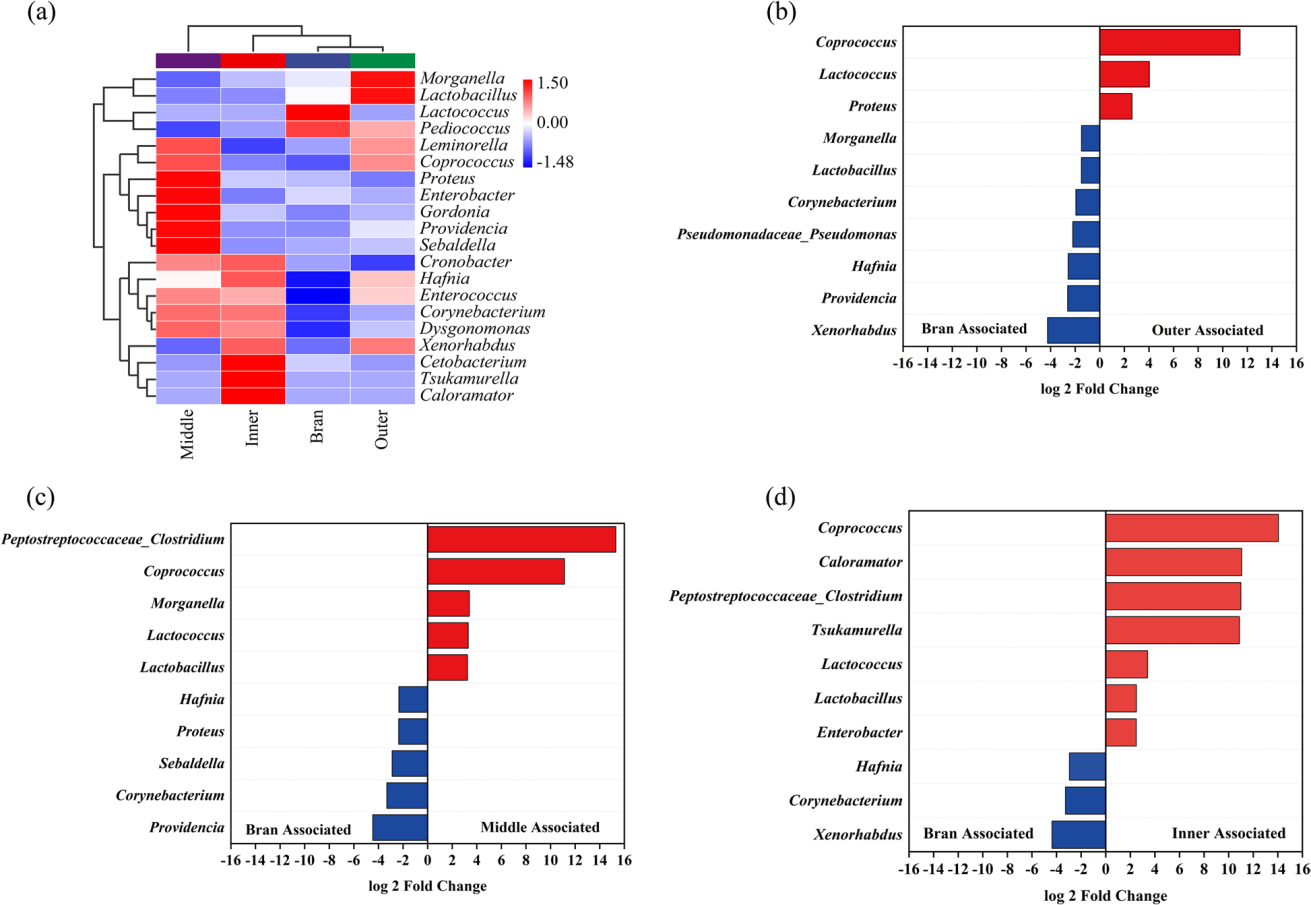
Significantly different bacteria at genus level. **a** Distribution of major genera (top 20); (**b**) Bran-fed vs. outer layer (**c**) Bran-fed vs. middle layer; (**d**) Bran-fed vs. inner layer

To further assess the significantly different bacteria and whether specific taxa were associated with different diets, a heat map of the microbial relative abundance at genus level was analyzed (Fig. 5a). The results showed that the number of significantly different taxa in the three layers was much higher than that in the WB group. A higher bacterial community indicated ecological stability and a microbial response to DFM. Functional bacteria associated with DFM degradation were also identified (Fig. 5b-d). It showed that the main functional bacteria for outer layer degradation were *Hafnia*, *Xenorhabdus*, and *Providencia*; *Xenorhabdus*, *Corynebacterium*, *Hafnia* for inner layer degradation; and *Hafnia*, *Providencia*, and *Corynebacterium* for middle layer degradation. The abundances of *Hafnia* and *Corynebacterium* were significantly higher, suggesting that these genera were significantly correlated with DFM-fed diet. Similarly, *Hafnia* has been shown to have plastic-degrading capabilities (Yang et al. 2019). In this study, they were associated with a PP diet in the gut microbiome of *Z. atratus* larvae (Fig. 4d). The genera *Corynebacterium* and *Pseudomonas* have been shown to be associated with the biodegradation of synthetic polymer materials (PE or PS) (Brandon et al. 2021; Tsochatzis et al. 2021; Yang et al. 2021c; Peng et al. 2022b; Wang et al. 2022a), and also showed significant differences in the *Z. atratus* group in relation to the PP diet in this study.

### Metabolome analysis

**Fig. 6:**
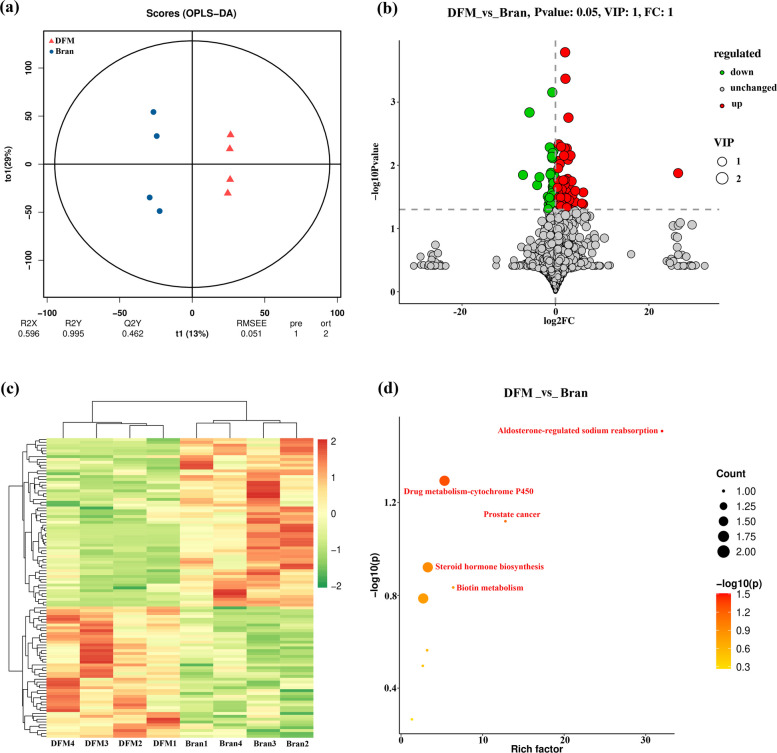
Gut metabolic expression in *Z. atratus* larvae. **a** OPLS-DA plots of metabolites in the DFM-fed and bran-fed groups (*n* = 4); **b** Differential metabolite categorization (*n* = 4); **c** Differential metabolite correlation heat maps (n = 4); **d** Differential metabolite bubble plots (*n* = 4)

To understand the metabolic pathways involved in DFM degradation by the gut microbiome, non-targeted metabolites of the gut contents were analyzed using liquid chromatography-mass spectrometry (LC–MS). The Orthogonal Partial Least Squares Discriminant Analysis (OPLS-DA) of metabolites showed two different clusters between the bran and DFM groups, suggesting enrichment of the DFM metabolic pathway of microbial degradation (Fig. 6a). According to the volcano plot analysis, metabolites differentially expressed in the gut of DFM-fed *Z. atratus*, of which 46 and 59 differentially-expressed metabolites were up- and downregulated, respectively (Fig. 6b). Hierarchical clustering analysis revealed similar results with significant differences between the two groups (Fig. 6c).

The results of the screening of these up-regulated differentially expressed metabolite pathways were showed in the bubble plot (Fig. 6d). Metabolic pathways, such as cytochrome P450, steroid hormone biosynthesis, and biotin metabolism, Aldosterone-regulated sodium reabsorption were significantly disrupted under DFM-feeding conditions. These metabolic pathways were associated with MP exposure. Comparative metabolomics analyses suggested that innate metabolic mechanisms in the gut coordinated with the redox capacity to synergize the biodegradation of DFM- polymers. These findings also suggested that *Z. atratus* utilized its endogenous reserves of metabolites to compensate for nutrient deficiencies and thus degraded DFM. Similar results have been reported for PS depolymerization/biodegradation (Sanchez-Hernandez 2021; Peng et al. 2022b; Chen et al. 2023; He et al. 2023; Byeon et al. 2024). This result was in line with the hypothesis of reported studies that reactive oxygen species were widely produced in the gut micro-environment of *Z. atratus* larvae as a result of multiple responses of intrinsic metabolic disorders, inflammation and the immune system, which facilitated the digestion of ingested biologically difficult to degrade polymers (Chen et al. 2023). To further support this result, the function genes of cytochrome P450, esterase and peroxidase based on 16S data were analyzed using PICRUSt2 (Fig. S3). It found that these genes were significantly enriched in the DFM-fed group, especially for esterase (*p* < 0.05) and peroxidase (*p* < 0.001). We hypothesized that despite the ability of *Z. atratus* to biodegrade DFM polymers, DFM ingestion reprogramed metabolic pathways in the gut environment, leading to in vivo metabolic dysregulation in *Z. atratus*, including oxidative stress induction. Consequently, the antioxidant systems and membranes were disrupted, and the energy supply of *Z. atratus* affected its growth. Therefore, further studies are required to test this hypothesis and understand the complex mechanisms by which MP-induced damage affects DFM biodegradation in *Z. atratus*.

In conclusion, the higher SR and CS of middle layer demonstrated the sufficient digestion and utilization of DFM middle layer by the larvae. Furthermore, the significant decrease in hydrophobicity and more large mass loss range showed the obvious enzymatic attack on C–C single bonds and the production of new organic matter. Moreover, changes in the chemical functional groups and increased oxygenated structures indicated the oxidization and depolymerization of DFM in the larval gut. These physicochemical changes further caused the obvious difference and cluster of microbial diversity between DFM-fed and WB-fed indicating the effects of DFM to the gut microbiome.

Changes in food can affect the characteristics of the gut microbial community. Therefore, the larvae ate and digested DFM caused the change in gut microbiome. Thus, the microbial diversity, composition and function changed, and further changed their metabolism. Finally, the oxidation and depolymerization of DFM happened namely the oxidation of PP.

### Degradation of DFM by Stenotrophomonas sp. strain M212

#### Isolation of DFM biodegradation strain

To further confirm the degradability of DFM by *Z. atratus* gut microbes, seven degrading strains were isolated by the enrichment of colonies formed in the larval gut (Fig. S4a). We then characterized biofilm formation on mask diaphragms to screen cultures for potential biodegradation. Biofilm formation allowed microorganisms to efficiently use insoluble substrates to characterize plastic-degrading strains (Kim et al. 2020; Peng et al. 2022a; Wang et al. 2022b; Nyamjav et al. 2023). Therefore, the isolated cultures were screened for potential DFM-degrading bacteria based on the number of cells in the biofilm colonizing the masks on the carbon-free basal agar medium (CFBAM) plates. Among the strains, the most abundant (9.7 ± 1.5 × 10^6^ CFU) (Fig. S4b) was taxonomically identified as *Stenotrophomonas* sp*.* strain M212 (Fig. S4c). *Stenotrophomonas acidaminiphila* had been found that showed PP-degrading ability (Jeyavani et al. 2024). This demonstrated that *Stenotrophomonas* sp. could degrade PP plastics, and therefore M212 was selected as a potential bacterial candidate for mask degradation for further study.

#### Characterization of bacterial biodegradable DFM

**Fig. 7:**
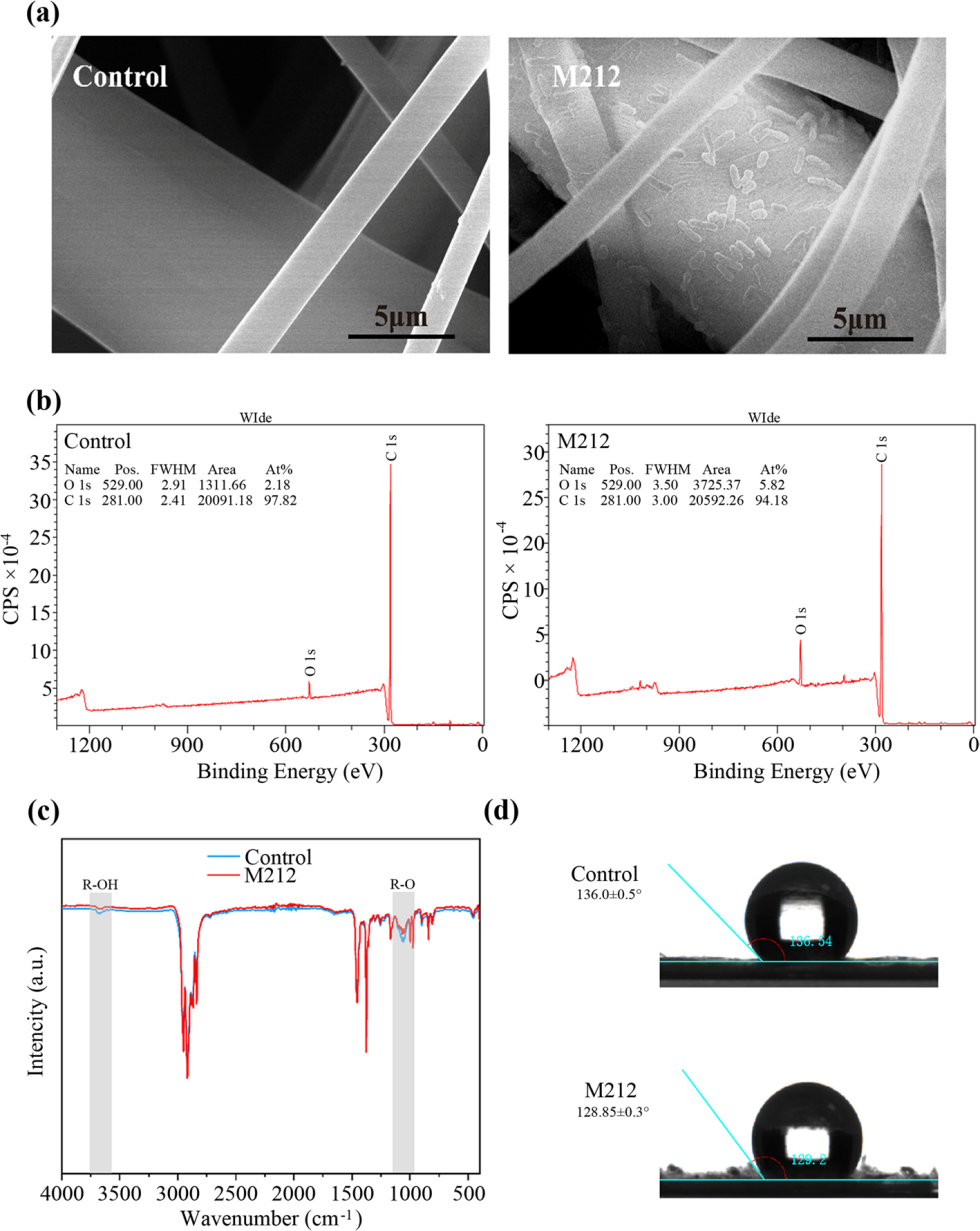
Surface chemical analysis of DFM samples from uninoculated DFM control and inoculated with strain M212. **a** SEM results of M212 degradation masks; (**b**) XPS scans, (**c**) FTIR scans and (**d**) decrease in WCA of residual DFM of uninoculated control and inoculated with M212 strain

Scanning Electron Microscope (SEM) observations (Fig. 7a) showed that M212 formed biofilms and exhibited significant degradation on the surface of the DFM with pits and cavities, This is similar to the degradation of PP plastic(Xian et al. 2023). The fiber surface of the un-inoculated control was smooth without any defects. The results showed that strain M212 disrupted the physical integrity of DFM.

X-ray Photoelectron Spectroscopy (XPS) and FTIR were used to analyze the changes in the surface chemistry and functional groups of DFM. Figure 7b showed the XPS scanning spectra (0–1200 eV) of DFM cultured with strain M212 versus an uncultured control sample. In the control sample, only surface carbon (284.8 eV) and surface oxygen (532.3 eV) were observed. The spectrum of the DFM sample containing strain M212 showed an increase in oxygen content from 2.18% to 5.82%. The spectrum of strain M212 showed a significant increase in oxygen content while the carbon content remained constant. The FTIR spectrum of strain M212 was compared with the control (Fig. 7c), it showed that peaks at 1260–1000 cm^−1^ (-C-O stretch) and the hydroxyl (-O–H) stretching absorption at approximately 3300–3600 cm^–1^ were both detected on DFM cultured with *Stenotrophomonas* sp*.* M212, suggesting the addition of oxygen during the biodegradation process. However, this was different from the degradation mechanism of other PP plastic-degrading bacteria. Appearance and disappearance of peaked in the carbonyl range of 1600–1850 cm^−1^ in the FTIR of bacterial strains treated with polypropylene microplastics indicating polymer degradation (Nanthini Devi et al. 2021; Xian et al. 2023; Jeyavani et al. 2024). These results suggested that strain M212 was capable of attacking or oxidizing DFM structures and producing more polar derivatives. Changes in surface hydrophobicity were analyzed using the WCA. After the complete removal of the formed biofilm from the mask samples, the WCA of the mask surface of strain M212 was 136.0° ± 0.5° (*n* = 3), which was lower than that of the control (128.9° ± 0.3°; *n* = 3; Fig. 7d). These results demonstrated that strain M212 also reduced the hydrophobicity of DFM with respect to biofilm formation. This decrease in hydrophobicity reduced the resistance of bacterial cells to subsequent degradation (Yang et al. 2015b).

## Conclusions

It firstly reported the DFM degradation by the gut microbiome of *Z. atratus* larvae. Mask depolymerization and biodegradation were achieved by oxidation and were gut microbe-dependent, with a high degrading rate of 60± 0.04 mg/d (dry mass by per 50 larvae) for the middle layer. DFM feeding altered the shift in functional gut microbiomes and, therefore, altered their metabolites. *Hafnia*, *Xenorhabdus*, and *Corynebacterium* were the potential mask-degrading bacteria. Furthermore, as identified by comparative metabolomics analyses, mask-degrading metabolite pathways were upregulated, including cytochrome P450, steroid hormone biosynthesis, and biotin metabolism. In addition, the regulatory P450 route could be mainly attributed to degradation. Moreover, *Stenotrophomonas* sp. M212 was screened from functional microbiomes with DFM-degrading ability, further confirming the oxidative degradation of DFM. Further studies should be conducted to (1) investigate whether the larva could rely on the energy gained from feeding on the DFM to maintain normal growth in the long term, (2) evaluate the individual role of the functional bacteria in DFM degradation, (3) and the key species for mask biodegradation and optimization of in vitro degradation capacity.

## Supplementary Information

Supplementary Material 1.

## Data Availability

All data generated or analyzed during this study are included in this published article [and its supplementary information files].

## Abbreviation

DFM: Disposable face mask
PP: Polypropylene
MPs: Microplastics
PS: Polystyrene
PE: Polyethylene
PVC: Polyvinyl chloride
PUR: Polyurethane reactive
WB: Wheat bran
WCA: Water contact angle
SEM: Scanning electron microscope
EDX: Energy dispersive X-ray spectroscopy
TGA: Thermogravimetric analysis
FTIR: Fourier-transform infrared spectroscopy
XPS: X-ray photoelectron spectroscopy
CR: Consumption rate
SR: Survival rate
ASVs: Amplicon sequence variants
OTUs: Operational taxonomic units
PCoA: Principal Coordinates Analysis
LC–MS: Liquid chromatography-mass spectrometry
OPLS-DA: Orthogonal partial least squares discriminant analysis
CFBAM: Carbon-free basal agar medium
FLASH: Fast length adjustment of short reads
LDPE: Low density polyethylene
TSA: Tryptic soy agar
