# Supplementary material for: Responses of gut microbial community and metabolic function to disposable face mask of Zophobas atratus larvae
Source: Adv Biotechnol (Singap). 2026 Jan 4;4(1):1. doi: 10.1007/s44307-025-00092-6 (PMC12765775; doi:10.1007/s44307-025-00092-6)
Supplement: Supplementary file 1 — Supplementary Material 1. [file 44307_2025_92_MOESM1_ESM.docx]

**Supplementary Information**

**Responses of microbial community and metabolic function to disposable face mask of the gut microbiome of Zophobas atratus larvae**

Chunlan Mao ^a,b1 *^, Kunyue Zhang ^c1^, Mamtimin Tursunay ^c^, Jing Ji^c^, Xiangkai Li ^c^

^a^ State Key Laboratory of Ecological Safety and Sustainable Development in Arid Lands, Northwest Institute of Eco-Environment and Resources, Chinese Academy of Sciences, Lanzhou 730000, China

^b^ Lanzhou Eco-Agriculture Experimental Research Station, Lanzhou 730000, China

^c^ Ministry of Education Key Laboratory of Cell Activities and Stress Adaptations, School of Life Science, Lanzhou University, Lanzhou, China

^1^ The author has equal contributions and is regarded as the co-first author.

^*^ Corresponding author: Chunlan Mao; E-mail address: maochunlan@nieer.ac.cn

**Journal name: Advanced Biotechnology**

**Contents:**

**1 Supplementary Text**

1.1 Text S1. Detailed experimental procedure of 16S rRNA sequencing

1.2 Text S2. Detailed experimental procedure of metabolome

1.3 Text S3. Screening and characterization of mask degrading competent isolates

**2 Supplementary Figures**

2.1 Fig S1. Photographs, SEM images, contact angles, and FTIR spectra of the outer, middle, and inner layers (from top to bottom) of DFMs.

2.2 Fig S2. Rarity curves based on the Shannon index of OTUs

2.3 Fig. S3. Enrichment of cytochrome P450, esterase and peroxidase genes on based on 16S data.

2.4 Fig S4. (a) Number of bacteria colonized on different strains isolated after 28 days of incubation on meltblown cloth film, (b) SEM plot of strain M212 (c) Phylogenetic tree of strain M212.

**3 Supplementary Table**

3.1 Table S1 Abundance of top 20 gut bacteria of *Z. atratus* larvae in the genera level

**Text S1 Detailed experimental procedure of 16S rRNA sequencing**

The total DNA of gut bacteria samples from *Z. atratus* fed with different diets were extracted using the E.Z.N.A.® soil DNA Kit (Omega Bio-tek, Norcross, GA, U.S.). The hypervariable region V3–V4 of the bacterial 16S rRNA gene were amplified by primers 338F (ACTCCTACGGGAGGCAGCAG) and 806R (GGACTACHVGGGTWTCTAAT). The resulting sequences were merged and filtered reads using FLASH (version 1.2.11) and fastp (version 0.19.6). Sequences < 50 bp in length with an average quality score < 20 and with ambiguous calls were discarded for subsequent analysis. Operational taxonomic units (OTUs) with 0.97 similarity cutoff were clustered using Uparse (version 11). The RDP Classifier (version 2.13) was used to analyze the taxonomy of each OTU representative sequence against the SILVA (version 138) 16s rRNA database using a confidence threshold of 0.7.

**Text S****2** **Detailed experimental procedure of metabolome**

Briefly, 50 mg of each collected whole intestinal tract tissue samples were firstly extracted using a 400 μL methanol:acetonitrile (1:1, V/V) solution containing internal standard of L-2-chlorophenylalanine (0.02 mg/mL). The extracted samples were placed at -20ºC for 30 min to precipitate the proteins after being treated by High throughput tissue crusher Wonbio-96c (Shanghai wanbo biotechnology co., LTD). Next, the extracted metabolites were centrifuged (13000 g, 15 min, 4^o^C) and the cleared supernatant were transferred to sample vials for LC-MS/MS analysis.

**Text S3 Screening and characterization of mask degrading competent isolates**

A group of 10 *Z. atratus* larvae were collected and fed with a mask as a single diet for 2 weeks to prepare an enterocyte suspension as an inoculum enriched for DMF-degrading bacteria as described above. The suspension was transferred to a 250 mL conical flask containing 1 g of small DMF flakes and 80 mL of LCFBM. The flask was incubated at ambient temperature on a rotary oscillator (120 rpm). After 60 days, residual DMF flakes were removed, and the enrichment was dispersed on plates with LB agar. After 24 hours of incubation at ambient temperature (22-24°C), colonies were picked and dispersed on other plates with fresh LB agar medium, where they were maintained until pure colonies of isolates were obtained based on morphologic observations of the colonies formed on the same plates and microscopic examination of cell morphology, To determine the bacterial species, genomic PCR was performed after extracting bacterial genomic DNA to obtain 16S RNA sequences with the following primers: forward primer 27-F (5′-AGTTTGATYMTGGGCTCAG-3′); reverse primer 1492-R (5′-GGTTACCTTGTTA-CGACTT-3′). 16S rRNA gene sequence analysis was performed to identify species. PCR was performed using a T100 Thermal Cycler and the amplified PCR DNA bands were analyzed by electrophoresis. After sequencing of the 16S rRNA DNA, bacterial species were identified using NCBI BLAST analysis.

**
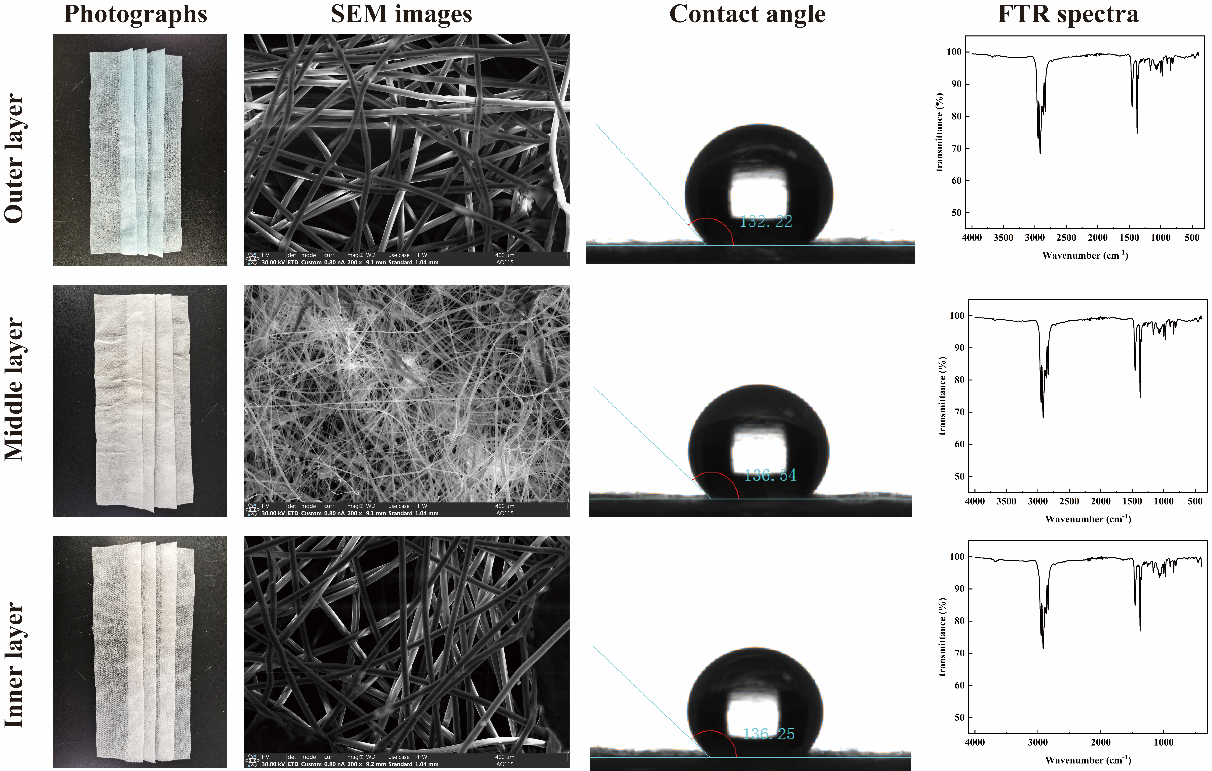
Fig. S1 Photographs, SEM images, contact angles, and FTIR spectra of the outer, middle, and inner layers (from top to bottom) of DFMs**

**
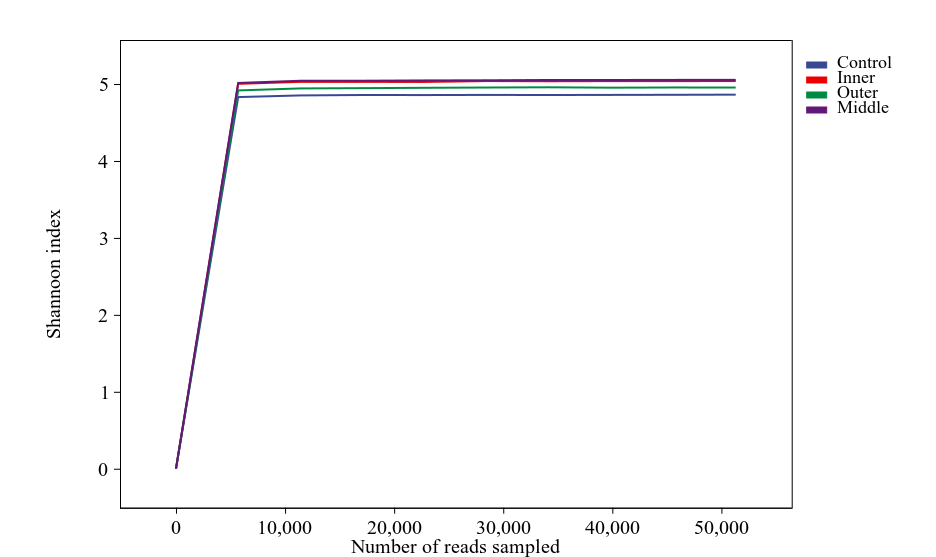
Fig. S2 Rarity curves based on the Shannon index of OTUs**


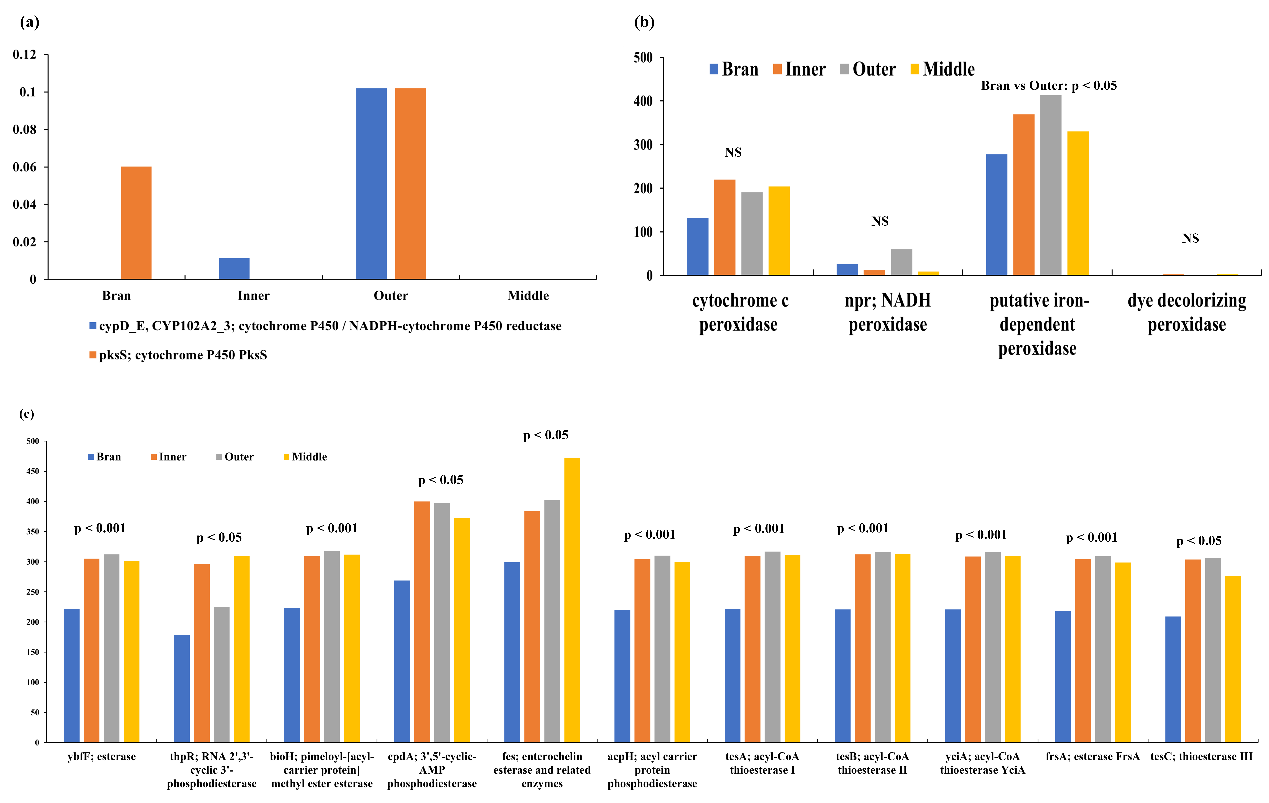


**Fig. S3 Enrichment of cytochrome P450, esterase and peroxidase genes on based on 16S data (n=3). NS: no significant**


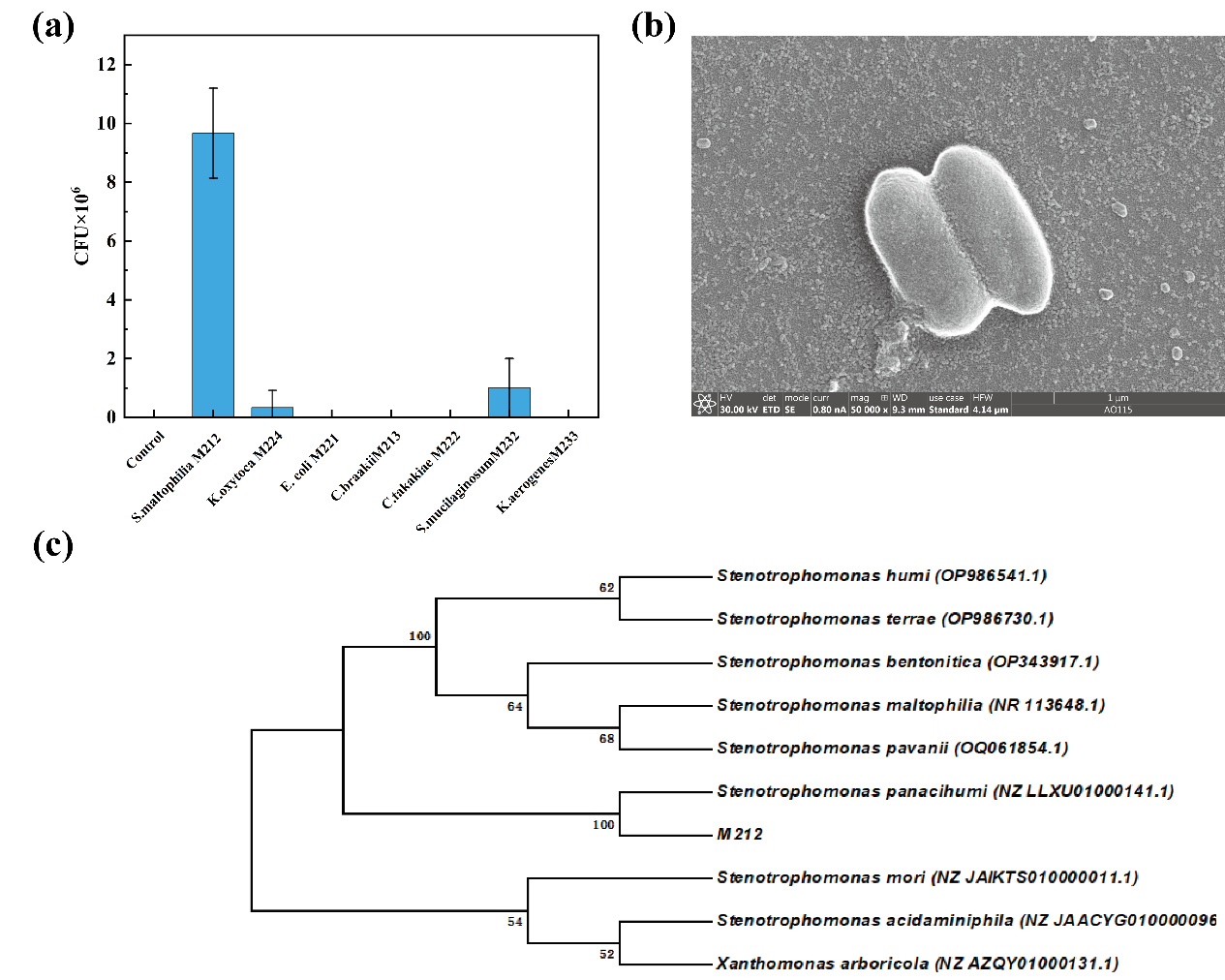
**Fig. S4 (a) Number of bacteria colonized on different strains isolated after 28 days of incubation on meltblown cloth film, (b) SEM plot of strain M212 (c) Phylogenetic tree of strain M212**

**Table S1 Abundance of top 20 gut bacteria of** ***Z. atratus* larvae in the genera level**

| ID | Bran | Inner | Outer | Middle |
| --- | --- | --- | --- | --- |
| Cronobacter | 9.84% | 17.91% | 6.76% | 16.55% |
| Lactococcus | 33.80% | 3.20% | 2.06% | 3.41% |
| Morganella | 5.49% | 3.90% | 15.42% | 0.52% |
| Enterococcus | 1.51% | 3.55% | 3.35% | 3.79% |
| Enterobacter | 1.46% | 0.26% | 0.91% | 5.26% |
| Lactobacillus | 1.74% | 0.31% | 4.91% | 0.18% |
| Hafnia | 0.27% | 2.12% | 1.62% | 1.38% |
| Pediococcus | 1.47% | 0.68% | 1.17% | 0.44% |
| Corynebacterium | 0.13% | 1.31% | 0.52% | 1.34% |
| Dysgonomonas | 0.20% | 0.62% | 0.40% | 0.67% |
| Providencia | 0.04% | 0.05% | 0.24% | 0.86% |
| Xenorhabdus | 0.02% | 0.38% | 0.34% | 0.01% |
| Proteus | 0.06% | 0.07% | 0.01% | 0.29% |
| Leminorella | 0.06% | 0.02% | 0.13% | 0.16% |
| Sebaldella | 0.03% | 0.01% | 0.04% | 0.19% |
| Pseudomonadaceae_Pseudomonas | 0.02% | 0.07% | 0.11% | 0.02% |
| Coprococcus | 0.00% | 0.01% | 0.04% | 0.04% |
| Tsukamurella | 0.00% | 0.05% | 0.00% | 0.00% |
| Peptostreptococcaceae_Clostridium | 0.00% | 0.05% | 0.00% | 0.00% |
| Caloramator | 0.00% | 0.05% | 0.00% | 0.00% |
| Others | 43.88% | 65.39% | 61.97% | 64.88% |
